# Mental Health Diagnoses Risk Among Children and Young Adults With Cerebral Palsy, Chronic Conditions, or Typical Development

**DOI:** 10.1001/jamanetworkopen.2024.22202

**Published:** 2024-07-19

**Authors:** Surbhi Bhatnagar, Alexis Mitelpunkt, Juliana J. Rizzo, Nanhua Zhang, Tess Guzman, Ryan Schuetter, Jilda Vargus-Adams, Amy F. Bailes, Kelly Greve, Melissa Gerstle, Ernest Pedapati, Bruce Aronow, Brad G. Kurowski

**Affiliations:** 1 Division of Biomedical Informatics, Cincinnati Children’s Hospital Medical Center, Cincinnati, Ohio; 2 Division of Pediatric Rehabilitation, Dana-Dwek Children’s Hospital, Tel Aviv Medical Center and Faculty of Medicine, Tel Aviv University, Tel Aviv, Israel; 3 College of Medicine, University of Cincinnati College of Medicine, Cincinnati, Ohio; 4 Division of Pediatric Rehabilitation Medicine, Cincinnati Children’s Hospital Medical Center, Cincinnati, Ohio; 5 Division of Biostatistics & Epidemiology, Cincinnati Children’s Hospital Medical Center, Cincinnati, Ohio; 6 Department of Pediatrics, University of Cincinnati College of Medicine, Cincinnati, Ohio; 7 Department of Neurology & Rehabilitation Medicine, University of Cincinnati College of Medicine, Cincinnati, Ohio; 8 Division of Occupational Therapy and Physical Therapy, Cincinnati Children’s Hospital Medical Center, Cincinnati, Ohio; 9 Department of Rehabilitation, Exercise and Nutrition Sciences, College of Allied Health Sciences, University of Cincinnati, Cincinnati, Ohio; 10 Division of Behavioral Medicine and Clinical Psychology, Cincinnati Children’s Hospital Medical Center, Cincinnati, Ohio; 11 Division of Child and Adolescent Psychiatry, Cincinnati Children’s Hospital Medical Center, Cincinnati, Ohio; 12 Division of Child Neurology, Cincinnati Children’s Hospital Medical Center, Cincinnati, Ohio

## Abstract

**Importance:**

Mental health (MH) issues in children with cerebral palsy (CP) are poorly understood compared with other pediatric populations.

**Objective:**

To examine MH diagnosis code assignment among children and young adults with CP and compare with typically developing (TD) and chronic condition (CC) pediatric populations.

**Design, Setting, and Participants:**

This case-control study used *International Statistical Classification of Diseases, Tenth Revision, Clinical Modification (ICD-10-CM)* codes to create a CP case set and CC and TD control sets using electronic health record data of children and young adults from a large tertiary care children’s hospital in the midwestern United States between 2010 and 2022. Case-control matching was performed to control for demographic factors. Data were analyzed from June to December 2023.

**Exposures:**

All MH diagnosis codes were mapped to *ICD-10-CM* and categorized using Clinical Classifications Software Refined (CCSR).

**Main Outcomes and Measures:**

The incidence rates of MH CCSR categories were calculated. Descriptive and comparative statistics were used to evaluate the significance and odds associated with factors.

**Results:**

Data from 216 794 individuals (mean [SD] baseline age, 4.3 [5.1] years; 118 562 [55%] male) were analyzed, including 3544 individuals with CP, 142 160 individuals with CC, and 71 080 TD individuals. The CP cohort spread across Gross Motor Function Classification System (GMFCS) levels I (981 individuals [28%]), II (645 individuals [18%]), III (346 individuals [10%]), IV (502 individuals [14%]), and V (618 individuals [17%]). Rates varied significantly for anxiety (824 individuals with CP [23%]; 25 877 individuals with CC [18%]; 6274 individuals with TD [9%]), attention-deficit/hyperactivity disorder (534 individuals with CP [15%]; 22 426 individuals with CC [16%]; 6311 individuals with TD [9%]); conduct or impulse disorder (504 individuals with CP [14%]; 13 209 individuals with CC [9%]; 3715 individuals with TD [5%]), trauma or stress disorders (343 individuals with CP [10%]; 18 229 individuals with CC [13%]; 5329 individuals with TD [8%]), obsessive-compulsive disorder (251 individuals with CP [7%]; 3795 individuals with CC [3%]; 659 individuals with TD [1%]), depression (108 individuals with CP [3%]; 12 224 individuals with CC [9%]; 4007 individuals with TD [5%]), mood disorders (74 individuals with CP [2%]; 4355 individuals with CC [3%]; 1181 individuals with TD [2%]), and suicidal ideation (72 individuals with CP [2%]; 7422 individuals with CC [5%]; 3513 individuals with TD [5%]). There was significant variation in odds of MH diagnoses by GMFCS level (I-II vs III-V: odds ratio [OR], 1.23; 95% CI, 1.09-1.40; *P* = .001). Among individuals with CP, males were more likely than females to have diagnosis codes for conduct or impulse disorders (OR, 1.41; 95% CI, 1.16-1.73) and attention-deficit/hyperactivity disorder (OR, 1.41 [95% CI, 1.15-1.73]). Black individuals, compared with White individuals, were more likely to have diagnoses for obsessive-compulsive disorder (OR, 1.57 [95% CI, 1.14-2.16]), other mood disorders (OR, 1.85 [95% CI, 1.01-3.38]), and trauma or stress disorders (OR, 1.94 [95% CI, 1.44-2.63]). Odds for trauma or stress disorders were elevated for individuals who identified as other races compared with White individuals (OR, 2.80 [95% CI, 2.03-3.87]).

**Conclusions and Relevance:**

In this case-control study of children and young adults with CP and matched comparisons, anxiety and conduct or impulse diagnoses were higher in individuals with CP. The lower diagnosis rates of depression and suicidal ideation may indicate underdiagnosis among individuals with CP. There is likely a need for assessment tools that are more suitable for children with CP.

## Key Points

**Question:**

Do rates of mental health diagnoses vary significantly within pediatric cerebral palsy (CP) populations and compared with other chronic conditions or typically developing pediatric populations?

**Findings:**

In this case-control study of 216 794 individuals aged 0 to 21 years with CP, chronic conditions, or typical development, there was marked variation in the rate of diagnosis code assignment within the CP cohort. The lower diagnosis rates of depression and suicidal ideation in the CP cohort may indicate underdiagnosis among individuals with CP, while anxiety and conduct or impulse disorder diagnoses were higher in the CP cohort.

**Meaning:**

These findings suggest that there is a need for mental health assessment tools that are suitable for children and young adults with CP.

## Teaser

This cohort study assesses mental health diagnosis code assignment among children and young adults with cerebral palsy and compared with matched typically developing and chronic condition populations.

## Introduction

Mental health (MH) in children and adolescents is a national health care crisis.^1,2,3,4,5^ Attention-deficit/hyperactivity disorder (ADHD), anxiety, behavior problems, and depression are the most common MH diagnoses and often occur comorbidly.^2^ MH in children with chronic conditions (CC) is not fully understood or characterized.^6,7,8,9^ Research indicates children with chronic physical conditions, such as cerebral palsy (CP), juvenile rheumatoid arthritis, cystic fibrosis, asthma, epilepsy, sickle-cell disease, spina bifida, and childhood cancers, are more likely to have a coexisting MH diagnosis.^9,10^ Given the medical and functional comorbidities associated with these chronic conditions, assessment and diagnosis of MH issues are challenging. Therefore, there is a gap in understanding of MH issues in many of these populations.

CP is unique compared with other CC, given the motor impairments associated with its diagnosis. CP is the most common physical disability in childhood, affecting approximately 1 in 323 children in the US.^11^ Children with CP are at a unique and elevated risk of behavioral and psychiatric problems because of their physical, social, communicative, and functional challenges.^12^ Nonetheless, there is wide variability in the reported rates of MH conditions in children with CP.^10,12,13,14,15^ Prior research largely explored convenience samples of children with CP, often in a relatively narrow age range, and lacked comparison groups.^10,12,14,15,16^ Descriptive data on the incidence of MH concerns in more extensive diverse populations of children with CP are limited. MH conditions may be unrecognized in people with CP due to the complexity of their disabilities and the need for assessment tools that accommodate differences in motor function and communication.^10,13^ Additionally, the diagnosis of MH disorders is challenging due to confounding factors, such as intellectual disability, communication barriers, or pain.^10^ Existing screening tools are not designed to assess MH in individuals with disabilities, emphasizing the need for diagnostic and screening tools tailored to CP.^12,13,16,17^

Using almost a decade of electronic health record (EHR) data, this project elucidates the use of MH diagnosis codes in children and young adults with CP compared with children and young adults with other CC and typically developing (TD) individuals. We hypothesized that patterns of MH diagnosis codes in individuals with CP would be similar to those in individuals with CC but would differ from patterns in TD individuals. We also hypothesized that MH diagnosis code rates would vary based on individual and CP-related factors, including race and ethnicity, sex, motor function, and age at initial CP diagnosis.

## Methods

This case-control study was approved by the Cincinnati Children's Hospital Medical Center institutional review board prior to the initiation of the study. Because this was a retrospective review of EHR data collected as part of standard clinical care, need for consent was waived. The study was conducted in adherence with the Strengthening the Reporting of Observational Studies in Epidemiology (STROBE) reporting guideline. We extracted EHR data from a large tertiary medical center in Ohio using the Observational Medical Outcomes Partnership Common Data Model.^18^ EHR data were obtained between January 1, 2010, and December 31, 2022, among individuals born after 1990 and aged 0 to 21 years at their first visit. We excluded individuals with fewer than 3 in-person visits to ensure sufficient interaction with the health care system. We used the *International Statistical Classification of Diseases, Tenth Revision, Clinical Modification (ICD-10-CM)* diagnosis codes to identify the CP cohort (eTable 1 in Supplement 1). We mapped all *International Classification of Diseases, Ninth Revision (ICD-9)* codes to *ICD-10-CM* codes using the General Equivalence Mapping for consistency. We created 2 control cohorts for comparison: individuals with CC included candidate controls with chronic respiratory, musculoskeletal or skin, neurological (excluding CP), and cardiovascular conditions, created using *ICD-10-CM* codes adapted from prior work,^19^ and TD individuals included candidate controls with none of the CP or CC diagnosis codes, seen in either sports medicine or emergency medicine, having 1 of the top 10 most frequently seen *ICD-10-CM* codes in the emergency department (eTable 1 in Supplement 1). We used the MatchIt package in R to perform case-control matching to account for demographic or individual factors that may introduce bias between the cohorts.^20^ We balanced the covariates age at the first visit in years, sex, race, ethnicity, and calendar year of the first visit by nearest-neighbor matching without replacement on propensity scores estimated using Mahalanobis distance with a caliper of 0.05 (eFigure 1 in Supplement 1). Demographics were well-documented since integration of the Epic EHR system (Epic Systems) in 2009. We selected data starting in 2010 to avoid any issues related to transition to the Epic EHR system. Race and ethnicity were missing for less than 1% of individuals. Date of birth and sex were documented for all patients in our cohort. The data dictionary is presented in Supplement 2.

The recorded Gross Motor Function Classification System (GMFCS) was used to stratify gross motor function in individuals with CP.^21^ We further mapped them to categories using the Clinical Classifications Software Refined (CCSR)^22^ categorization (eTable 2 in Supplement 1). The deprivation index measures the level of deprivation in an area (range, 0-1; higher score indicated greater deprivation) and was calculated using methods described in prior work.^23^ Race and ethnicity were examined among the groups to assess disparities in MH diagnosis and incidence. The individual or guardian reported both race and ethnicity. Race was categorized as Asian, Black, multiracial, White, or other (races observed in <1% of patients, including American Indian or Alaskan Native, Middle Eastern or North African, Native Hawaiian or Other Pacific Islander, and race not listed). Ethnicity was categorized as Hispanic or non-Hispanic.

### Statistical Analysis

Our primary analysis was exploratory, where MH diagnoses in individuals with CP were compared with those in CC and TD cohorts. We also examined factors associated with different MH diagnoses as a secondary exploratory analysis.

Descriptive and comparative statistics were used to evaluate the significance and odds associated with factors. We used the χ^2^ test for large samples and Fisher exact test for small samples (<5) to examine the independence of categorical variables. Cohort differences of continuous variables were calculated using the Wilcoxon rank sum test. Since both analyses were exploratory, no adjustment of the significance threshold was made. However, a stringent 2-sided *P* ≤ .01 was used because of the large sample size and multiple outcomes. We calculated odds ratios (ORs) of patient characteristics associated with MH conditions based on independent logistic regression models. All analyses used Python version 3.11 (Python Software Foundation, R version 4.1.2 (R Project for Statistical Computing), or SAS version 9.4 (SAS Institute).

## Results

We analyzed EHR data from 216 794 individuals (mean [SD] age at first visit, 4.3 years [5.1] years; 118 562 [55%] male), including 3554 individuals with CP, 142 160 individuals with CC, and 71 080 TD individuals. The characteristics of the final cohorts after matching are described in Table 1. The mean (SD) age across all visits was 8.4 (5.7) years (median [IQR], 8 [3-13] years) across all patient visits. A total of 9377 individuals were Hispanic and 206 212 individuals were non-Hispanic, and 1205 individuals were of unknown ethnicity. A total of 5369 individuals were Asian, 34 612 individuals were Black, 7779 individuals were multiracial, 161 039 individuals were White, 4943 individuals identified as other race, and 3052 individuals were of unknown race. The CP cohort was spread across the spectrum of GMFCS motor function, with 9981 individuals (28%) with level I, 645 individuals (18%) with level II, 346 individuals (10%) with level III, 502 individuals (14%) with level IV, and 618 individuals (17%) level V. Most individuals in the CP cohort also had neurodevelopmental disorders, as typically seen in individuals with CP, particularly speech (2124 individuals [60%]), motor (1871 individuals [53%]), and pervasive (327 individuals [9%]) developmental disorders. A total of 558 individuals with CP (16%) also had an intellectual disability diagnosis. The most common MH conditions identified were anxiety, ADHD, conduct or impulse disorders, trauma or stress disorders, obsessive-compulsive disorder (OCD), depression, mood disorders, and suicidal ideation or attempts.

**Table 1.** Characteristics of Cerebral Palsy, Chronic Conditions, and Typically Developing Cohorts

Characteristic	Individuals, No. (%)		
	Cerebral palsy (n = 3554)	Chronic condition (n = 142 160)	Typically developing (n = 71 080)
Sex			
Male	1979 (56)	76 387 (54)	40 196 (56)
Female	1575 (44)	65 773 (46)	30 884 (44)
Age at first visit, y			
Mean (SD)	4.3 (5.2)	4.4 (5.2)	4.1 (5.1)
Median (IQR)	2 (0-8)	2 (0-8)	2 (0-7)
Age at first MH diagnosis, y			
Mean (SD)	9.4 (4.8)	10.5 (4.6)	10.5 (4.4)
Median (IQR)	9 (5-13)	10 (7-14)	11 (7-14)
Age at CP diagnosis, y			
Mean (SD)	5.1 (4.9)	NA	NA
Median (IQR)	3 (1-8)	NA	NA
Ethnicity			
Hispanic	173 (5)	6371 (5)	2833 (4)
Non-Hispanic	3361 (95)	135 056 (95)	67 795 (95)
Unknown	20 (<1)	733 (<1)	452 (<1)
Race			
Asian	108 (3)	3476 (3)	1785 (3)
Black	531 (15)	22 724 (16)	11 357 (16)
Multiracial	127 (3)	4820 (3)	2832 (4)
White	2638 (74)	105 929 (75)	52 472 (74)
Other^a^	91 (3)	3262 (2)	1590 (2)
Unknown	59 (2)	1949 (1)	1044 (1)
Deprivation index, mean (SD)^b^	0.35 (0.11)	0.35 (0.11)	0.35 (0.11)

Abbreviations: CP, cerebral palsy; MH, mental health; NA, not applicable.

^a^ Includes American Indian or Alaskan Native, Middle Eastern or North African, Native Hawaiian or other Pacific Islander, and race not listed.

^b^ The deprivation index measures the level of deprivation in an area with a range of 0 to 1 and a higher score for greater deprivation.

**Figure 1.**
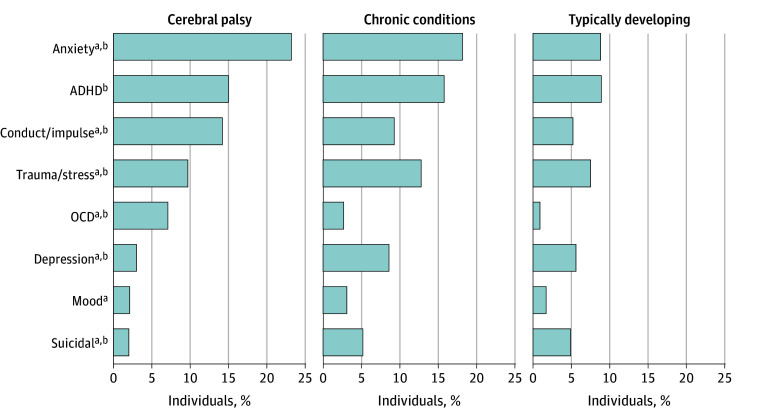
Mental Health Diagnosis Code Assignment in Cerebral Palsy, Chronic Conditions, Typically Developing Cohorts Anxiety includes anxiety and fear-related disorders; conduct/impulse, disruptive, impulse-control, and conduct disorders; trauma/stress, trauma- and stressor-related disorders; depression, depressive disorders; mood, other specified and unspecified mood disorders; suicidal, suicidal ideation or attempt, or intentional self-harm. ADHD indicates attention-deficit/hyperactivity disorder; OCD, obsessive-compulsive and related disorders. ^a^Indicates condition significantly differed (*P* < .01) between cerebral palsy and chronic condition groups. ^b^Indicates condition significantly differed between cerebral palsy and typically developing groups.

We computed the rates of diagnosis code assignment with each MH code among the CP cohort. We observe a marked difference in the proportion of diagnosis codes of MH conditions between individuals in CP and both control cohorts (Figure 1). The differences in diagnosis rates between CP and 1 or both comparison cohorts were significant for anxiety (824 individuals with CP [23%]; 25 877 individuals with CC [18%]; 6274 individuals with TD [9%]), ADHD (534 individuals with CP [15%]; 22 426 individuals with CC [16%]; 6311 individuals with TD [9%]); conduct or impulse disorder (504 individuals with CP [14%]; 13 209 individuals with CC [9%]; 3715 individuals with TD [5%]), trauma or stress disorders (343 individuals with CP [10%]; 18 229 individuals with CC [13%]; 5329 individuals with TD [8%]), obsessive-compulsive disorder (251 individuals with CP [7%]; 3795 individuals with CC [3%]; 659 individuals with TD [1%]), depression (108 individuals with CP [3%]; 12 224 individuals with CC [9%]; 4007 individuals with TD [5%]), mood disorders (74 individuals with CP [2%]; 4355 individuals with CC [3%]; 1181 individuals with TD [2%]), and suicidal ideation (72 individuals with CP [2%]; 7422 individuals with CC [5%]; 3513 individuals with TD [5%]). Individuals with CP, compared with both control cohorts, were more likely to have anxiety (OR vs CC, 1.27 [95% CI, 1.18-1.37]; OR vs TD, 2.59 [95% CI, 2.39-2.81]), ADHD (OR vs CC, 0.96 [95% CI, 0.88-1.06]; OR vs TD, 1.72 [95% CI, 1.57-1.90]), conduct or impulse disorders (OR vs CC, 1.57 [95% CI, 1.43-1.72]; OR vs TD, 2.80 [95% CI, 2.54-3.08]), trauma or stress disorders (OR vs CC, 0.79 [95% CI, 0.70-0.88]; OR vs TD, 1.35 [95% CI, 1.20-1.50]), OCD (OR vs CC, 2.89 [95% CI, 2.54-3.27]; OR vs TD, 8.60 [95% CI, 7.44-9.92]), depression (OR vs CC, 0.35 [95% CI, 0.29-0.43]; OR vs TD, 0.54 [95% CI, 0.44-0.65]), mood (OR vs CC, 0.68 [95% CI, 0.53-0.86]; OR vs TD, 1.25 [95% CI, 0.97-1.59]), and suicidal ideation or attempt (OR vs CC, 0.39 [95% CI, 0.31-0.49]; OR vs TD, 0.41 [95% CI, 0.32-051]). Mental health conditions, including ADHD, anxiety, trauma or stress disorders, and OCD, were observed at significantly higher rates in individuals with CP compared with TD. However, depression and suicidal ideation or attempts were observed to be markedly lower in the CP group. Compared with individuals with CC, individuals with CP had significantly higher diagnosis rates for anxiety, conduct or impulse disorders, and OCD. Depression and suicidal ideation were observed at significantly lower rates.

**Figure 2.**
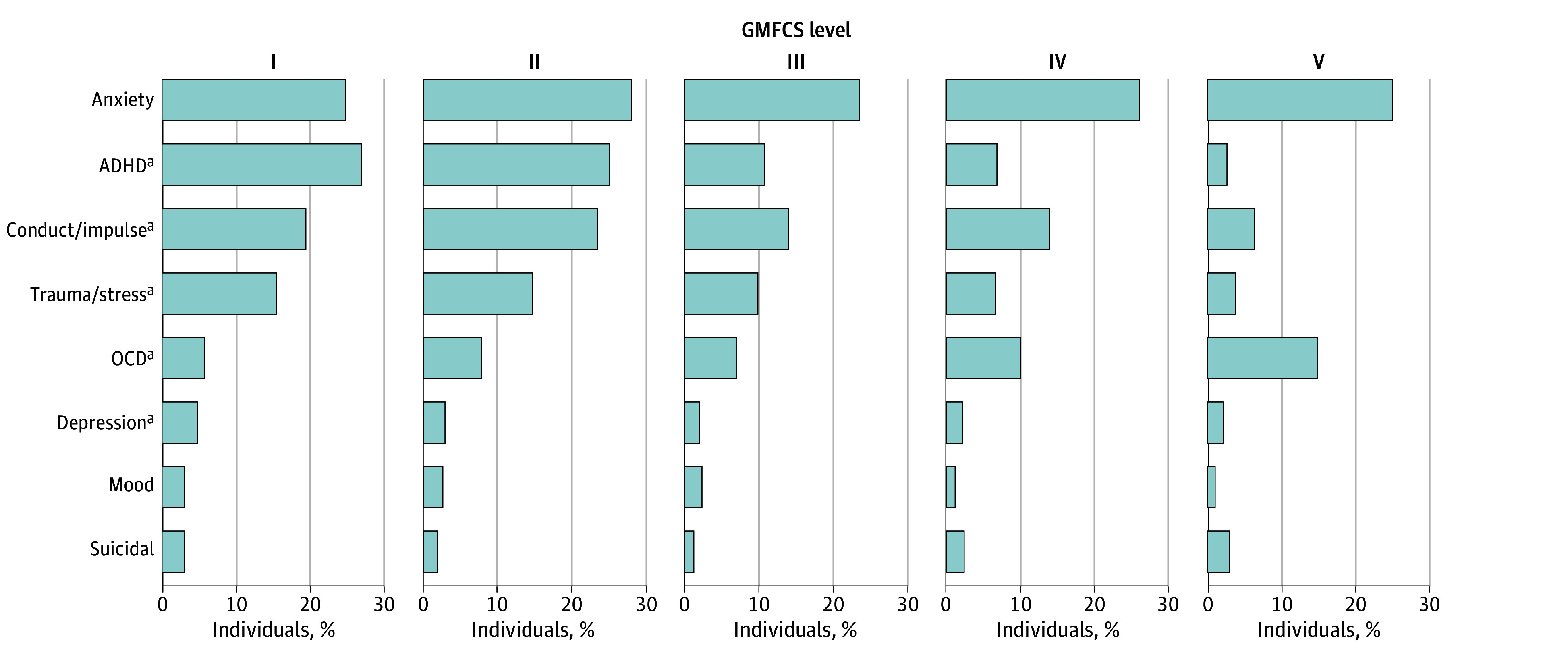
Mental Health Diagnosis Code Assignment in Cerebral Palsy Cohort by Gross Motor Function Classification System (GMFCS) GMFCS level I (981 participants [28%]) indicates walks without limitations; II (645 participants [18%]), walks with limitations; III, (346 participants [10%]), walks using a handheld mobility device; IV (n = 502 participants [14%]), self mobility with limitations, may use powered mobility; and V (618 participants [17%]), transported in a manual wheelchair. ^a^Indicates condition significantly differed across the GMFCS levels (*P* < .01; χ^2^ test). Anxiety includes anxiety and fear-related disorders; depression, depressive disorders; conduct/impulse, disruptive, impulse-control, or conduct disorders; trauma/stress, trauma- and stressor-related disorders; mood, other specified and unspecified mood disorders; suicidal, suicidal ideation or attempt, or intentional self-harm. ADHD indicates attention-deficit/hyperactivity disorder; OCD, obsessive-compulsive and related disorders.

To examine diagnosis code assignment rates among individuals with different gross motor functions in the CP cohort, we examined MH by the documented GMFCS levels (Figure 2). Notably, we observed significantly higher odds of diagnosis for ADHD, conduct or impulse disorders, trauma or stress disorders, and depression across all GMFCS levels, with the diagnosis rate decreasing for higher GMFCS levels, indicating an association with less significant motor impairment. OCD exhibited the reverse association, with higher rates of diagnosis found among participants with higher GMFCS levels, suggesting that more significant motor impairment was associated with OCD. Furthermore, GMFCS level was associated with significantly varied risk for most MH conditions. Compared with GMFCS level I, higher GMFCS levels were associated with lower odds for the development of ADHD, conduct or impulse disorders (except GMFCS II vs I), depression, mood, and trauma or stress disorders and higher odds for the development of OCD (Table 2).

**Table 2.** Odds of Mental Health Diagnoses Among Children and Young Adults With Cerebral Palsy by Demographic Factors, Deprivation Index, and GMFCS

Factor	OR (95% CI)							
	Disorder							Suicidal ideation or attempt or intentional self-harm (n = 72)
	Attention-deficit/hyperactivity (n = 534)	Anxiety and fear-related (n = 824)	Disruptive, impulse-control, conduct (n = 504)	Depressive (n = 108)	Other specified and unspecified mood (n = 74)	Obsessive-compulsive and related (n = 251)	Trauma- and stressor- related (n = 343)	
GMFCS level								
I	1 [Reference]	1 [Reference]	1 [Reference]	1 [Reference]	1 [Reference]	1 [Reference]	1 [Reference]	1 [Reference]
II	0.92 (0.73-1.16)	1.19 (0.95-1.50)	1.36 (1.07-1.74)^a^	0.53 (0.30-0.91)^a^	0.83 (0.45-1.54)	1.38 (0.93-2.05)	0.89 (0.67-1.19)	0.60 (0.30-1.19)
III	0.32 (0.22-0.47)^a^	0.94 (0.71-1.26)	0.70 (0.50-0.99)^a^	0.35 (0.16-0.79)^a^	0.71 (0.32-1.58)	1.18 (0.72-1.95)	0.57 (0.38-0.86)^a^	0.37 (0.13-1.06)
IV	0.20 (0.13-to 0.29)^a^	1.09 (0.85-1.40)	0.71 (0.53-0.96)^a^	0.36 (0.18-0.71)^a^	0.35 (0.14-0.86)^a^	1.67 (1.11-2.50)^a^	0.36 (0.24-0.54)^a^	0.76 (0.38-1.52)
V	0.07 (0.04-to 0.11)^a^	1.01 (0.80-1.28)	0.29 (0.20-0.42)^a^	0.34 (0.18-0.65)^a^	0.28 (0.12-0.69)^a^	2.69 (1.89-3.83)^a^	0.20 (0.13-0.32)^a^	0.93 (0.51-1.70)
Age at CP diagnosis, per 1-y increase	1.00 (0.98-to 1.02)	1.00 (0.98-1.01)	0.97 (0.95-0.99)^a^	1.10 (1.06-1.14)^a^	1.07 (1.02-1.12)^a^	1.03 (1.01-1.06)^a^	1.04 (1.01-1.06)^a^	1.04 (0.99-1.08)
Sex								
Male (vs female)	1.41 (1.15-1.73)^a^	1.00 (0.84-1.17)	1.41 (1.16-1.73)^a^	0.69 (0.46-1.04)	0.67 (0.41-1.10)	1.20 (0.93-1.55)	0.82 (0.65-1.03)	0.95 (0.60-1.51)
Race								
Black	1.08 (0.82-1.44)	1.05 (0.84-1.32)	1.29 (0.99-1.69)	1.44 (0.85-2.43)	1.85 (1.01-3.38)^a^	1.57 (1.14-2.16)^a^	1.94 (1.44-2.63)^a^	1.11 (0.59-2.09)
White	1 [Reference]	1 [Reference]	1 [Reference]	1 [Reference]	1 [Reference]	1 [Reference]	1 [Reference]	1 [Reference]
Other^b^	0.97 (0.69-1.36)	0.86 (0.64-1.15)	0.96 (0.69-1.36)	0.98 (0.48-2.00)	1.42 (0.66-3.07)	0.85 (0.52-1.37)	2.80 (2.03-3.87)^a^	1.33 (0.65-2.72)
Deprivation index^c^	0.61 (0.24-1.52)	0.62 (0.29-1.32)	0.65 (0.26-1.61)	1.97 (0.32-12.17)	0.19 (0.02-2.10)	0.75 (0.23-2.40)	1.39 (0.48-3.99)	1.56 (0.20-12.18)

Abbreviations: CP, cerebral palsy; GMFCS, Gross Motor Function Classification System; OR, odds ratio.

^a^ Indicates significantly different MH conditions (*P* < .01).

^b^ Includes American Indian or Alaskan Native, Asian, Middle Eastern or North African, multiracial, Native Hawaiian or other Pacific Islander, and race not listed.

^c^ The deprivation index measures the level of deprivation in an area with a range of 0 to 1 and a higher score for greater deprivation. ORs were calculated on a continuous scale.

**Figure 3.**
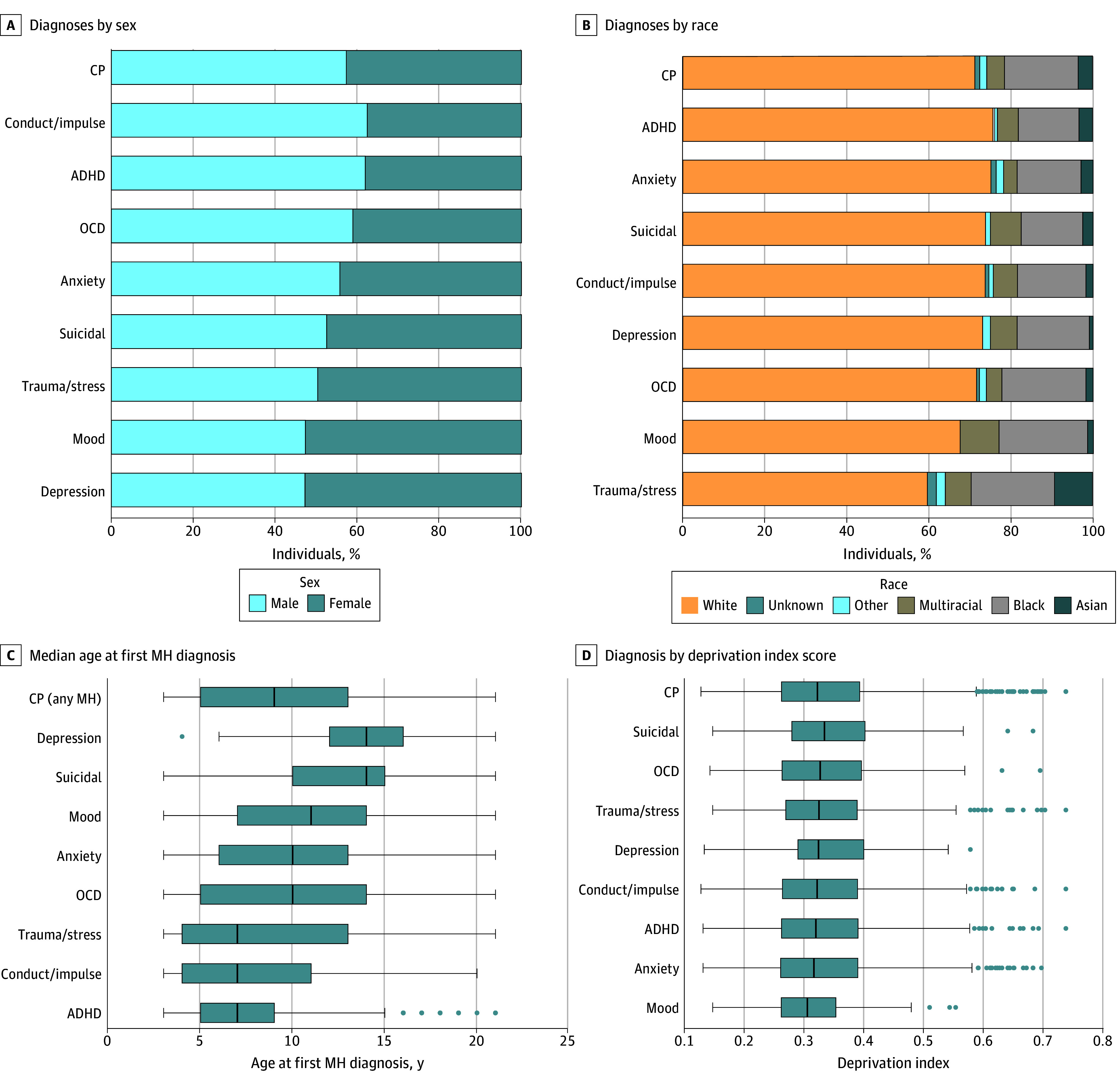
Distribution of Mental Health Diagnosis Code Assignment in the Cerebral Palsy (CP) Cohort by Sex, Race, Age at First Use of a Mental Health (MH) Diagnosis Code, and Deprivation Index Score The other race category includes Middle Eastern or North African, Native Hawaiian or other Pacific Islander, American Indian or Alaskan Native, and race not listed. The deprivation index measures the level of deprivation in an area, with a higher score for greater deprivation. Anxiety includes anxiety and fear-related disorders; depression, depressive disorders; conduct/impulse, disruptive, impulse-control, or conduct disorders; trauma/stress, trauma- and stressor-related disorders; mood, other specified and unspecified mood disorders; suicidal, suicidal ideation or attempt, or intentional self-harm. ADHD indicates attention-deficit/hyperactivity disorder; OCD, obsessive-compulsive and related disorders. Thick lines indicate median; bars, IQRs; whiskers, ranges; dots, individual data points.

Additionally, we examined variations in the rates of MH diagnosis by sex, race, number of visits, and age at initial MH diagnosis (Figure 3). Among individuals with CP, males were more likely than females to have diagnosis codes for conduct or impulse disorders (OR, 1.41; 95% CI, 1.16-1.73) and ADHD (OR, 1.41; 95% CI, 1.15-1.73). We observed higher odds of diagnosis in the Black population compared with the White population in the CP group for OCD (OR, 1.57; 95% CI, 1.14-2.16), other mood disorders (OR, 1.85; 95% CI, 1.01-3.38) and trauma or stress disorders (OR, 1.94; 95% CI, 1.44-2.63). We also observed higher rates of trauma or stress among the Asian population in the CP cohort (OR, 4.34; 95% CI, 2.85-6.61]; *P* < 001). Odds for trauma or stress disorders were elevated for individuals who identified as other race compared with White individuals (OR, 2.80; 95% CI, 2.03-3.87) (Table 2).

## Discussion

To our knowledge, this case-control study is the first study to use more than a decade of EHR data from a tertiary care pediatric center to characterize the use of MH-related diagnosis codes in individuals with CP and compare them with matched groups. This study fills a critical gap in the field by providing an understanding of MH diagnosis rates in children and young adults with CP compared with TD individuals and children and young adults with other CC. A 2019 cross-sectional study^12^ in a smaller CP population (111 individuals) found that there were higher odds of MH problems, specifically anxiety and behavior or conduct disorders, compared with rates in the general population.^2^ Also, a 2020 population-based study of caregivers from a Swedish CP register who completed screening questionnaires found higher positive screening results for autism spectrum disorder and ADHD than already identified in the caregivers’ children.^14^ Our study expands on prior work by providing unique, longitudinal insight into the variation of a broader range of MH diagnosis code use across GMFCS levels, race, ethnicity, deprivation index, and sex of individuals with CP. This study provides vital information on the burden of MH in children and young adults with CP. The most common MH diagnoses in individuals with CP were anxiety, ADHD, conduct or impulse disorders, trauma or stress disorders, and OCD. Furthermore, ADHD was more common in the CP than in the general population.^13^ Psychiatric diagnoses that encompass emotional and behavioral issues are common, affecting more than half of school-aged children with CP.^10,15,24^

Anxiety, conduct or impulse disorders, and OCD were diagnosed more frequently in CP compared with both TD and CC control populations. These higher rates may be due to the physical challenges defining CP or their social, communicative, and functional implications.^12,25^ Individuals with CP are at a unique and elevated risk of behavioral and psychiatric problems.^12,26^ Findings from a 2019 cohort study suggest that adults with CP are at higher risk of anxiety and depression, especially in the absence of intellectual disability.^27^ The uptick in MH diagnoses could also result from inaccurate ascertainment or mischaracterization of MH symptoms for children with CP, perhaps due to challenges in symptom recognition due to these same functional and communication limitations. Given the range of gross motor abilities across GMFCS levels in children with CP, motor responses might be misinterpreted as signs or symptoms of externalizing behavior issues, leading to diagnostic errors. Ongoing efforts should explore optimal mechanisms to identify MH concerns across all CP subgroups. Future research should evaluate the reasons behind higher rates of anxiety, conduct or impulse disorders, and OCD in CP using comprehensive qualitative studies to understand precise causes and establish optimal identification and management approaches for children with CP.

Depression is an ongoing MH concern in the pediatric CP population, with some studies reporting higher rates than in general populations.^16,28^ Factors such as pain, family functioning, fatigue, and the impact of the COVID-19 pandemic contribute to depression and anxiety for many individuals.^16,28,29^ However, in this study, individuals with CP had significantly lower rates of diagnosis of depression and suicidal ideation or attempts compared with both TD and other CC populations, indicating either a lower prevalence of these conditions in individuals with CP, underrecognition of the symptoms associated with these conditions, or lack of formal diagnosis.

Identifying and diagnosing MH conditions may be more challenging in children with CP due to motor, communication, intellectual, or functional impairments; thus, children with such difficulties may struggle to express feelings of sadness, respond to standard screening questionnaires for depression, or describe atypical manifestations of depression, such as a lack of interest in typical activities or lack of appetite. Some symptoms of depression may be mistaken for side effects of medications (eg, malaise due to sedation from antispasticity or antiepileptic drugs) or consequences of physical impairment (eg, limited activity). Additional research is needed to evaluate the reasons for these lower diagnosis rates and to establish valid assessment and diagnostic tools. Further evaluation is also required to assess whether depression or suicidal ideation is being misdiagnosed as anxiety, trauma or stress, conduct or impulse disorders, or other behavioral conditions we observe at higher rates in CP.

This study also underscores the association of individual and CP-related factors, including sex, race, presence of intellectual impairment, and GMFCS level, with variation in rates of MH diagnosis. ADHD, conduct or impulse disorders, and trauma or stress disorders had the most pronounced variation across GMFCS levels. The diagnosis rates were inversely correlated with GMFCS levels, with the higher GMFCS levels (reflecting more significant motor impairment) having much lower diagnosis rates. This association may be related to difficulty in assessing MH symptoms in children with CP with higher GMFCS levels who may exhibit higher rates of gross motor functional impairments or comorbid communication or intellectual disability. Rates of MH diagnosis varied across race and ethnicity. We observed significantly higher odds of mood, OCD, and trauma or stress among Black individuals compared with White individuals with CP. Prior work has highlighted the need for better MH screening and access for Black populations.^30^ Males had a marked increase in odds for ADHD and conduct or impulse disorders compared with females, which is similar to prior research.^31^ We observed that older age at CP diagnosis was significantly associated with decreased odds of conduct or impulse disorders and with increased odds of depression, mood, OCD, and trauma or stress disorders. Compared with GMFCS level I, higher GMFCS levels were associated with lower odds of developing ADHD, conduct or impulse disorder (except GMFCS II vs I), depression, mood disorders, and trauma or stress disorders and with higher odds of developing OCD. A 2018 systematic review and meta-analysis^32^ found no association between age and motor function; however, children with intellectual disability were observed to have a higher risk of MH symptoms. Proactive screening for MH conditions should be tailored based on GMFCS levels and other demographic factors.

### Limitations

This study has some limitations. We relied on clinician-assigned *ICD-10-CM* codes in the EHR for diagnostic classification rather than formal psychological or neuropsychological evaluation. This includes the formal classification of intellectual disability, which is a significant risk factor for MH concerns.^32^ It is likely that rates of intellectual disability codes are underused because these codes were not the primary reason for a clinical encounter. Some variation may be added due to updated coding standards, since the *ICD-9* codes were mapped to *ICD-10-CM* for consistency, and *Diagnostic and Statistical Manual of Mental Disorders* (Fifth Edition) (*DSM-5*) was implemented during this period.^33^ The lack of appropriate MH diagnostic tools specific to children with CP may limit the ability of clinicians to identify MH conditions accurately or expeditiously.^32^ The study’s retrospective design inherently restricts the ability to control for confounding variables and biases, as is possible with a prospective design. Since this study was exploratory in nature, strict multiple testing correction was not used, which increases the chance of type I error. This study cannot account for the variation in MH diagnosis codes used across health care practitioners. Still, the findings highlight the need to develop better ways to assess MH to pursue appropriate management and treatment. Furthermore, because data represent a single midwestern US tertiary medical center, rates reported may not be generalizable to other geographic locations or types of centers. Also, due to the broad age range of participants included, rates of MH diagnoses may be underrepresented overall, especially since MH diagnoses increase with age into adolescence and young adulthood.^2^ Although we reported deprivation index ranges as a marker of socioeconomic status for this population, we do not have specific information on maternal education available. Future research should evaluate the reasons behind the observed variation and establish optimal diagnostic tools and approaches. For our future work, we plan to improve our understanding of mental health risk factors, experience, environment, and resilience for better identification by leveraging clinical progress notes using natural-language processing and artificial intelligence–based models.

## Conclusions

This case-control study offers a novel characterization of MH diagnosis rates in children and young adults with CP within a large EHR-based study, providing insights into MH burden and diagnostic patterns. These findings suggest that MH diagnoses were prevalent in CP, varied based on CP motor function and other demographic factors, and may have been underrecognized or overrecognized, depending on the MH diagnosis. Acknowledging the signs of MH issues in children is crucial, as early intervention can significantly improve both MH and CP treatment engagement and lead to better outcomes. Accurate recognition and diagnosis of MH issues in children with CP are essential for providing effective interventions and improving overall quality of life.

## Notes

Supplement 1. **eTable 1.** Cohort Creation Method Using *International Statistical Classification of Diseases and Related Health Problems* (*ICD*) Diagnosis Codes **eTable 2.** Mental Health Conditions of Interest and Number of People per Condition in the CP Cohort **eFigure 1.** Covariate Balance Before and After Matching Using Propensity Score for the Cerebral Palsy and Chronic Conditions Cohort and the Cerebral Palsy and Typically Developing Cohort **eFigure 2.** Distribution of Mental Health Diagnosis Code Assignment in the Cerebral Palsy Cohort by Number of Visits Having an MH Diagnosis Code

Supplement 2.
Data Dictionary

Supplement 3.
Data Sharing Statement
