# Errors in Results Sections of Abstract and Main Text

**DOI:** 10.1001/jamanetworkopen.2024.60923

**Published:** 2025-01-22

In the Original Investigation titled “Mental Health Diagnoses Risk Among Children and Young Adults With Cerebral Palsy, Chronic Conditions, or Typical Development,”^1^ published July 19, 2024, the percentages given for the chronic conditions and typically developing cohorts were transposed in the Results sections of the Abstract and main text. This article has been corrected.^1^
