# Supplementary material for: Mental Health Diagnoses Risk Among Children and Young Adults With Cerebral Palsy, Chronic Conditions, or Typical Development
Source: JAMA Netw Open. 2024 Jul 19;7(7):e2422202. doi: 10.1001/jamanetworkopen.2024.22202 (PMC11259902; doi:10.1001/jamanetworkopen.2024.22202)
Supplement: Supplement 1. — eTable 1. Cohort Creation Method Using International Statistical Classification of Diseases and Related Health Problems (ICD) Diagnosis Codes eTable 2. Mental Health Conditions of Interest and Number of People per Condition in the CP Cohort eFigure 1. Covariate Balance Before and After Matching Using Propensity Score for the Cerebral Palsy and Chronic Conditions Cohort and the Cerebral Palsy and Typically Developing Cohort eFigure 2. Distribution of Mental Health Diagnosis Code Assignment in the Cerebral Palsy Cohort by Number of Visits Having an MH Diagnosis Code [file jamanetwopen-e2422202-s001.pdf]

Supplemental Online Content

Bhatnagar S, Mitelpunkt A, Rizzo JJ, et al. Mental health diagnoses risk among children and young adults with cerebral palsy, chronic conditions, or typical development. JAMA Netw Open. 2024;7(7):e2422202. doi:10.1001/jamanetworkopen.2024.22202

eTable 1. Cohort Creation Method Using International Statistical Classification of Diseases and Related Health Problems (ICD) Diagnosis Codes

eTable 2. Mental Health Conditions of Interest and Number of People per Condition in the CP Cohort

eFigure 1. Covariate Balance Before and After Matching Using Propensity Score for the Cerebral Palsy and Chronic Conditions Cohort and the Cerebral Palsy and Typically Developing Cohort

eFigure 2. Distribution of Mental Health Diagnosis Code Assignment in the Cerebral Palsy Cohort by Number of Visits Having an MH Diagnosis Code

This supplemental material has been provided by the authors to give readers additional information about their work.

© 2024 Bhatnagar S et al. *JAMA Network Open*.

Etable 1: Cohort creation method using International Statistical Classification of Diseases and Related Health Problems (ICD) diagnosis codes

Cohort	Methods/ICD Codes used
Cerebral Palsy (CP) n=3,554	Patients with at least one of the following CP diagnostic codes.<br>ICD10CM codes: G80.0, G80.1, G80.2, G80.3, G80.4, G80.8, G80.9, G81.00, G81.01, G81.02, G81.04, G81.10, G81.11, G81.12, G81.13, G81.14, G81.90, G81.91, G81.92, G81.93, G81.94<br>ICD9Diag codes: 342.00, 342.01, 342.02, 342.10, 342.11, 342.12, 342.80, 342.81, 342.82, 342.9, 342.90, 342.91, 342.92, 343.0, 343.1, 343.2, 343.3, 343.4, 343.8, 343.9, 344.0, 344.00, 344.01, 344.02, 344.03, 344.04, 344.09, 344.1, 344.2, 344.30, 344.40, 344.41, 344.5, 344.60, 344.61, 344.89, 344.9
Chronic Condition (CC) n=142,160	All chronic respiratory, musculoskeletal/skin, neurological (excluding CP), and cardiovascular conditions ICD codes listed in Wijlaars et al. [20]
Typically Developing (TD) n=71,080	Patients who visit either Sports Medicine (SM) or Emergency Medicine (EM) with one of the top 10 most frequently seen diagnostic codes in the EM department and none of the CP or CC diagnosis codes.<br>Top EM ICD10CM codes: E86.0, J06.9, K59.00, R05, R05.1, R05.2, R05.3, R05.4, R05.8, R05.9, R09.81, R10.9, R11.10, R19.7, R45.851, R50.9, R51, R51.0, R51.9, Z20.822, Z32.02, Z81.8

© 2024 Bhatnagar S et al. *JAMA Network Open*.

Etable 2: Mental Health Conditions of Interest and Number of people per condition in the CP cohort.

Condition Label	Condition Description	Included codes CCSR/ICD10	CP n=3,554
Anxiety	Anxiety and fear-related disorders	MBD005	824
ADHD	Attention-deficit/ hyperactivity disorder	F90	534
Conduct/ Impulse	Disruptive, impulse-control, conduct disorders	MBD008	504
Trauma/ Stress	Trauma- and stressor-related disorders	MBD007	343
OCD	Obsessive-compulsive and related disorders	MBD006	251
Depressio n	Depressive disorders	MBD002	108
Mood	Other specified and unspecified mood disorders	MBD004	74
Suicidal	Suicidal ideation/ attempt/intentional self-harm	MBD027, MBD012	72

MH: Mental Health conditions. The corresponding CCSR or ICD10CM codes for each condition are listed. The ICD10CM Codes also include all the children of the specific ICD code. ICD10CM: International Statistical Classification of Diseases and Related Health Problems version 10 Clinical Modification, CCSR: Clinical Classifications Software Refined.

© 2024 Bhatnagar S et al. *JAMA Network Open*.

Efigure 1: Covariate Balance before and after matching using propensity score for A) the Cerebral Palsy and Chronic Conditions (CP-CC) cohort and B) the Cerebral Palsy and Typically Developing (CP-TD) cohort.

A)

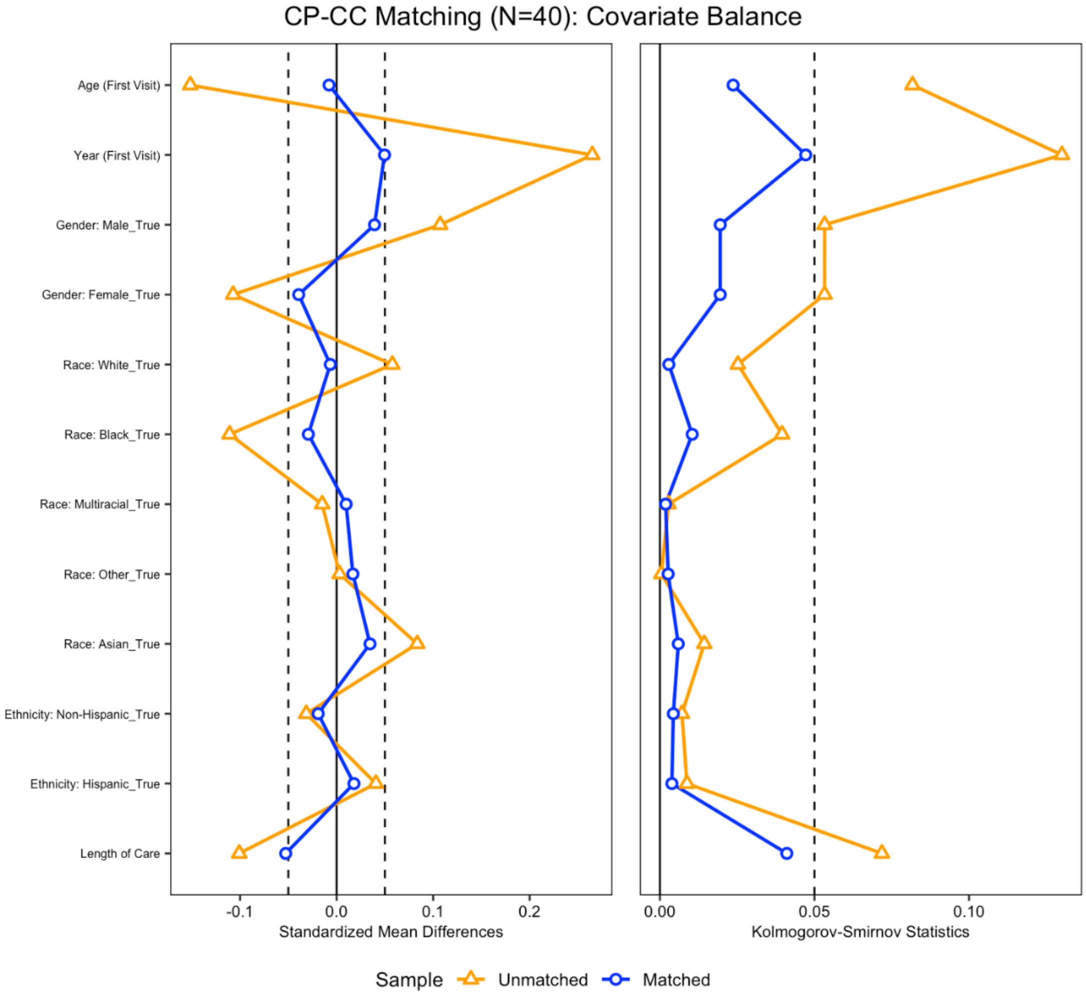

B)

© 2024 Bhatnagar S et al. *JAMA Network Open*.

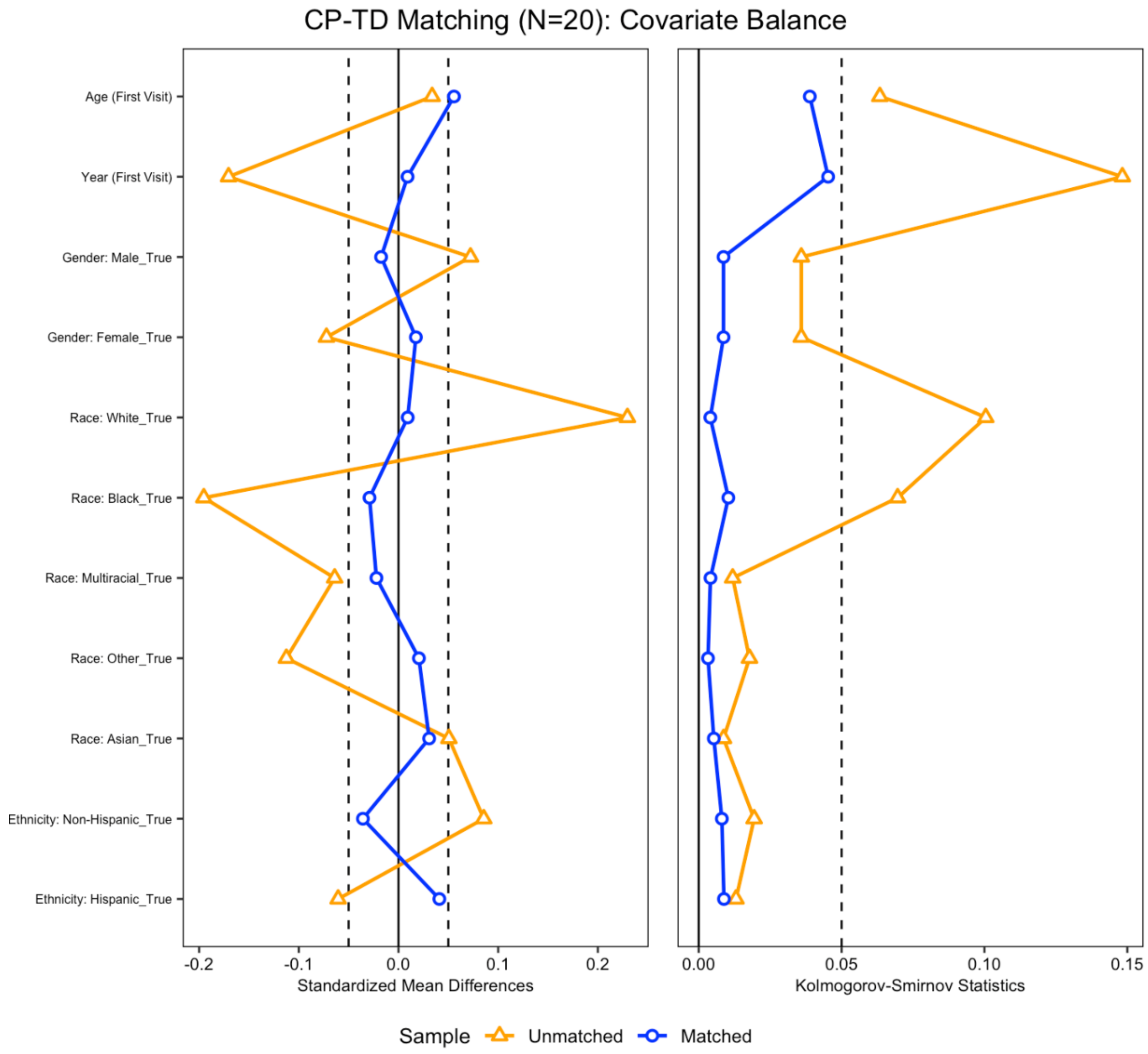

The balance is shown before and after matching for the covariates: age at the first visit, sex, race, ethnicity, and calendar year of the first visit. The MatchIt package was used in R to perform case-control matching to account for demographic or individual factors that may introduce bias between the cohorts. We used nearest neighbor matching without replacement on propensity scores estimated using Mahalanobis distance with a caliper of 0.05. CP: Cerebral Palsy (n=3,554), CC: Chronic Condition (n=142,160), TD: Typically Developing (n=71,080).

© 2024 Bhatnagar S et al. *JAMA Network Open*.

Efigure 2: Distribution of Mental Health Diagnosis Code Assignment in the Cerebral Palsy Cohort by Number of Visits having an MH Diagnosis Code.

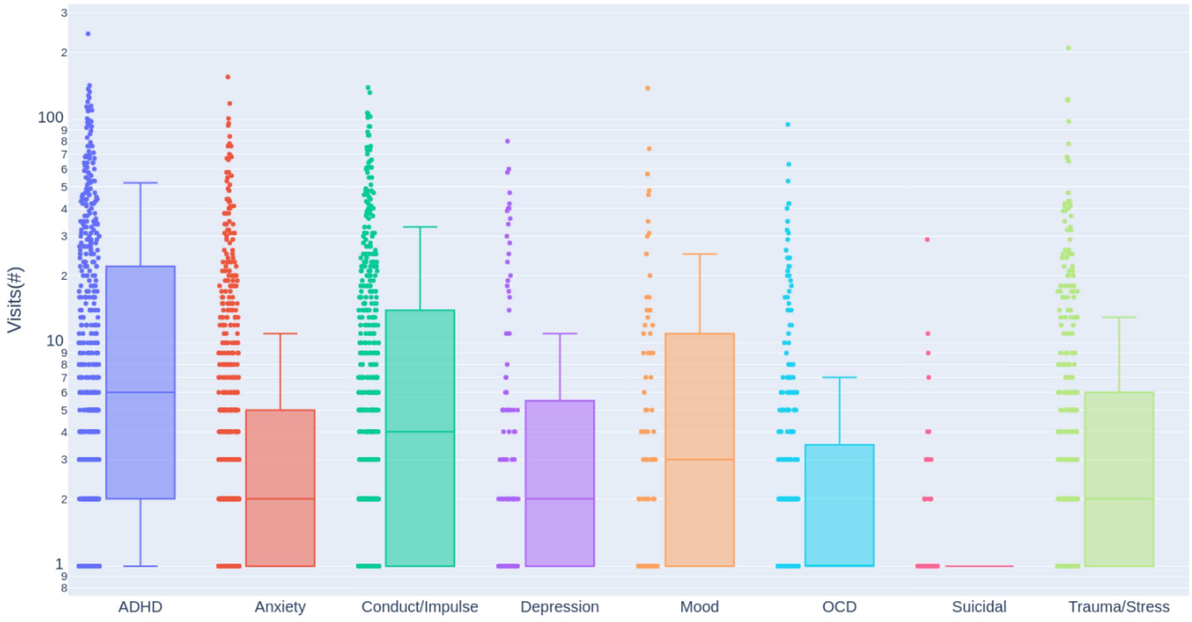

The distribution of the number of visits is colored by the MH condition. The number of visits is shown on a log 10 scale. Anxiety: Anxiety and fear-related disorders. ADHD: Attention-deficit hyperactivity disorder, Conduct/Impulse: Disruptive, impulse-control, conduct disorders. Trauma/Stress: Trauma- and stressor-related disorders. OCD: Obsessive-compulsive and related disorders. Depression: Depressive disorders. Mood: Other specified and unspecified mood disorders. Suicidal: Suicidal ideation/attempt/intentional self-harm.

© 2024 Bhatnagar S et al. *JAMA Network Open*.
