# Supplementary material for: Mental Health Diagnoses Risk Among Children and Young Adults With Cerebral Palsy, Chronic Conditions, or Typical Development
Source: JAMA Netw Open. 2024 Jul 19;7(7):e2422202. doi: 10.1001/jamanetworkopen.2024.22202 (PMC11259902; doi:10.1001/jamanetworkopen.2024.22202)
Supplement: Supplement 3. — Data Sharing Statement [file jamanetwopen-e2422202-s003.pdf]

Data Sharing Statement

Bhatnagar. Mental Health Diagnoses Risk Among Children and Young Adults With Cerebral Palsy, Chronic Conditions, or Typical Development. *JAMA Netw Open*. Published July 19, 2024. doi:10.1001/jamanetworkopen.2024.22202

Data
Data available: Yes
Data types: Data dictionary
How to access data: Send requests to brad.kurowski@cchmc.org
When available: With publication

Supporting Documents
Document types: Other (please specify)
Additional Information: Supporting data dictionary
How to access documents: Request to brad.kurowski@cchmc.org
When available: With publication

Additional Information
Who can access the data: Researchers whose proposed use of data has been approved.
Types of analyses: Specified purpose
Mechanisms of data availability: With investigator support, after approval of proposal, and/or signed data access agreement.
